# Loss of OprD function is sufficient for carbapenem-resistance-only but insufficient for multidrug resistance in *Pseudomonas aeruginosa*

**DOI:** 10.1186/s12866-025-03935-3

**Published:** 2025-04-16

**Authors:** Maofeng Wang, Yuxiao Zhang, Fengyan Pei, Ying Liu, Yan Zheng

**Affiliations:** 1 Research Center of Translational Medicine, Central Hospital Affiliated to Shandong First Medical University, Jinan, Shandong China; 2 Medical Research & Laboratory Diagnostic Center, Central Hospital Affiliated to Shandong First Medical University, Jinan, Shandong China

**Keywords:** *Pseudomonas aeruginosa*, Carbapenem resistance, OprD inactivation, Porin deficiency

## Abstract

**Background:**

Carbapenem-resistant *Pseudomonas aeruginosa* (CRPA) constitutes a serious source of global healthcare-associated infections, and the exploration of its resistance mechanism represents an important approach to address this issue. Because current research on antibiotic resistance predominantly focuses on multidrug-resistant *P. aeruginosa* which is widely isolated clinically and the resistance mechanism is complicated. CRPA generally has a higher tolerance to other antibiotics than carbapenem-sensitive *P. aeruginosa*, yet the specific mechanism of resistance remains poorly understood.

**Results:**

This study delves into the specific antibiotic resistance mechanisms of carbapenem-resistance-only *P. aeruginosa* (CROPA), a rare kind of pathogen that shows resistance exclusively to carbapenem antibiotics. We collected 11 clinical isolates of CROPA, performed genome sequencing. Our analysis revealed numerous amino acid mutations and premature termination of OprD expression in the CROPA strains. The insertion of IS256 element into OprD in *P. aeruginosa* was a novel finding. Validation via qPCR and SDS-PAGE affirmed diminished OprD expression levels. Interestingly, common carbapenemases were not detected in our study, and there was no observed upregulation of relevant efflux pumps. The expression of wild-type OprD in CROPA strains restored the sensitivity to carbapenem antibiotics.

**Conclusions:**

Compared with previous studies on MDR-CRPA, the emergence of CROPA may be directly linked to changes in OprD, while other resistance mechanisms could contribute to broader antibiotic resistance profiles. By focusing on the antibiotic resistance mechanisms of CROPA, this study illuminates the relationship between specific antibiotic resistance mechanisms and antibiotic resistance, providing a theoretical foundation for guiding clinical treatment and developing novel anti-infective agents.

**Supplementary Information:**

The online version contains supplementary material available at 10.1186/s12866-025-03935-3.

## Highlights

The focal point of this study rests on the uncommon CROPA strain.

Compared with MDR-CRPA, the development of CROPA is directly linked to the inactivation of OprD.

Reviving OprD enhances CROPA’s susceptibility to carbapenems.

**Supplementary Information:**

The online version contains supplementary material available at 10.1186/s12866-025-03935-3.

## Introduction

*Pseudomonas aeruginosa* is a commonly encountered opportunistic pathogen in hospital settings that can cause both acute and chronic infections in immunocompromised individuals, such as those with chronic obstructive pulmonary disease, cystic fibrosis, cancer, wound, burns, sepsis, and ventilator-associated pneumonia, including infections caused by COVID-19 [1–3]. Traditional antibiotic therapy is still predominantly used to treat *P. aeruginosa* infections [4]. Carbapenems, renowned for their potent antibacterial activity, enzyme stability, and minimal adverse effects, are frequently employed in managing severe clinical infections [5], particularly those caused by *P. aeruginosa* [6, 7]. The mechanism of action of carbapenems involves penetration into bacteria through porins, inhibition of peptidoglycan synthesis enzymes, disruption of cell wall synthesis, leading to bacterial cell lysis, and eventual death [8, 9].

Resistance of *P. aeruginosa* to carbapenems, such as imipenem, meropenem, or ertapenem, is defined as carbapenem resistance *P. aeruginosa* (CRPA) [10, 11]. CRPA generally has a higher tolerance to other antibiotics than carbapenem-sensitive *P. aeruginosa* [12]. The issue of carbapenem resistance in *P. aeruginosa* is becoming increasingly critical, with most CRPA strains exhibiting resistance mediated by multiple mechanisms [13, 14]. Resistance to carbapenems also extends to other classes of antibiotics, making the treatment of CRPA infections progressively challenging [15].

Current research on multidrug-resistant CRPA (MDR CRPA) has identified various resistance mechanisms [16], including reduced outer membrane (OprD) permeability [17], altered penicillin-binding proteins (PBPs), increased efflux [18], β-lactamase hydrolysis [19], and biofilm formation [20]. Efforts have been made by researchers to identify new antibiotic targets and modify antibiotics to enhance sensitivity. However, the complex and varied nature of resistance mechanisms in MDR CRPA, involving numerous genetic mutations and regulatory mechanisms, in combination with resistance mechanisms to other antibiotics, complicates the elucidation of specific genetic or regulatory factors responsible for carbapenem resistance [21–24].

In our clinical observations, we have identified a small number of carbapenem-resistant-only *P. aeruginosa* (CROPA) strains (11/492) that are sensitive to all tested anti-*Pseudomonas* antibiotics (penicillins, cephalosporins, monobactams, quinolones, aminoglycosides, and lipopeptides) but resistance to carbapenems (imipenem, meropenem, or both) [25]. Given that CRPA strains typically exhibit resistance to other antibiotics as well [26], unraveling the interplay between multidrug resistance mechanisms in CRPA and carbapenem resistance poses a formidable challenge. Therefore, exploring the resistance mechanisms of CROPA could deepen our understanding of the relationship between resistance mechanisms and antibiotic resistance.

This study started with 11 clinical isolates of CROPA and used genomic sequencing technology to reveal numerous gene mutations. And the influence of gene mutations on resistance was verified by qPCR, SDS-PAGE, and antibiotic susceptibility testing. Through comparison with MDR-CRPA, it was found that the emergence of CROPA may be mainly related to changes in OprD, while other resistance mechanisms may contribute to a broader spectrum of antibiotic resistance. By obtaining specific resistance conclusions, this study aims to discover the best treatment strategy to enhance therapeutic effectiveness.

## Method

### Isolation and identification of bacteria along with antimicrobial susceptibility testing

Clinical samples were collected from a tertiary hospital in Shandong Province between 2021 and 2023, excluding duplicate bacterial strains from the same patient in brief period. The specimens were inoculated onto Columbia agar plates and placed in an incubator. After 24 h of incubation at 35 °C, 5% CO_2_, colonies exhibiting round, variable sizes, flat, raised, smooth, moist, and metallic sheen characteristics, suspected to be *P. aeruginosa*, were selected using a specialized loop and streaked onto detection target plates for identification using the Bruker MALDI-TOF-MS.

Following the identification of *P. aeruginosa* colonies via mass spectrometry, purified isolates were cultured in Mueller-Hinton (MH) medium for 24 h and subjected to antimicrobial susceptibility testing using the VITEK 2 COPACT automated microbial sensitivity analysis system (BioMérieux, France), employing Gram-negative bacterial sensitivity cards N335. The interpretation of antimicrobial susceptibility test results was conducted according to the 2023 Clinical and Laboratory Standards Institute (CLSI) standards (CLSI M100 ED33-2023) [27]. Control strains used, as provided by the National Health Commission Clinical Testing Center included *P. aeruginosa* ATCC 27853 and *Escherichia coli* ATCC 25922. The tested antibiotics encompassed penicillins, cephalosporins, monobactams, quinolones, aminoglycosides, carbapenems, and lipopeptides. The susceptibility profiles of *P. aeruginosa* to these antibiotics — categorized as sensitive, intermediate, or resistant — were summarized. CRPA was defined as resistance to either imipenem or meropenem, both carbapenems. CROPA exhibiting resistance only to carbapenems while remaining sensitive to other drugs were selected for further investigation into their resistance mechanisms.

### Carbapenemase determination

The search was conducted on NCBI to identify known β-lactamases (VIM, IMP, KPC-2, OXA-48, NDM-1) in *P. aeruginosa*, with primers (Table S1) based on established literature [28]. DNA templates, including genomic and plasmid DNA, were obtained using a boiling method, and *rpsL* served as a positive control to verify the availability of those templates. Following PCR amplification, the presence of known β-lactamases was assessed [29].

### Whole-genome sequencing and data analysis

The 11 CROPA were cultured in LB medium to OD_600_ = 0.8 for DNA extraction. Libraries were constructed using the Illumina TruSeqTM Nano DNA Sample Prep Kit method, followed by paired-end sequencing of the DNA samples using Illumina NovaSeq6000 sequencing technology. The ABySS v2.0.2 software (http://www.bcgsc.ca/platform/bioinfo/software/abyss) was utilized for sequence assembly, optimizing the sequences with multiple Kmer parameters to achieve the most favorable assembly outcomes. Subsequently, the GapCloser v1.12 software (https://sourceforge.net/projects/soapdenovo2/files/GapCloser/) was employed for local gap filling and base correction on the assembled results. Predicted gene protein sequences were subjected to blastp comparisons against the NR, Swiss-Prot, eggNOG, KEGG, and GO databases (BLAST + 2.7.1, alignment criteria: E-value ≤ 1e-5) to acquire annotation information for the predicted genes. We aligned these sequences against the protein sequences of *P. aeruginosa* PAO1, and the corresponding reference sequence accessions are as follows: *ftsI* (GenBank: WKE25042.1), *oprD* (GenBank: CAA78448.1), *mexR* (GenBank: WKE26597.1), *nalC* (GenBank: AAG07108.1), *nalD* (GenBank: AAG06962.1), *nfxB* (GenBank: WKE25217.1), *mexS* (GenBank: WKE28644.1), *mexZ* (GenBank: AAC64520.1), *wspF* (GenBank: WKE24330.1), *rpoS* (GenBank: WKE24250.1), *wapR* (GenBank: AAG08385.1), *galU* (GenBank: WKE28182.1), *algU* (GenBank: AAG04151.1), *and rpoN* (GenBank: AAG07850.1). This alignment helped to identify any variations or mutations in these specific genes compared to the reference genome.

Multilocus sequence typing (MLST) was conducted following the PubMLST protocol (http://pubmlst.org/paeruginosa/).

### RNA isolation and real-time quantitative PCR (RT-qPCR)

The expression of genes encoding efflux pump proteins *mexA*, *mexC*, *mexE*, and *mexX*, as well as the o*prD* porin gene, was analyzed using RT-qPCR following the previously described protocol [30]. Primer sequences for RT-qPCR are provided in Table S2. Total RNA was extracted using a MiniBEST Universal RNA Extraction Kit (TaKaRa, Beijing, China), and cDNA synthesis was performed using a PrimeScript RT reagent Kit (TaKaRa, Beijing, China). RT-qPCR was carried out using 2× SYBR Green qPCR Mix (Sparkjade®, Shandong, China) with gene-specific primers (see Table S2) on an Applied Biosystems QuantStudio 1 instrument (Thermo Fisher Scientific, MA, US). The expression of the 30 S ribosomal gene *rpsL* was used to normalize the transcript levels of the target genes. A reduction in o*prD* expression of ≥ 50% compared to the *P. aeruginosa* strain PAO1 was considered significant. Each isolate was tested three times, and the average value was reported as the result of gene expression.

### Construction of the *oprD* knockout strain and complemented strain

Gene deletion mutants of *P. aeruginosa* PAO1 were created via Redγ-BAS recombinase-mediated homologous recombination. Primers used for gene deletions are listed in Table S2, incorporating ∼ 75 bp homology arms flanking the target gene for cloning the gentamicin resistance gene cassette with a LoxP site from the template plasmid pR6K. Electroporation of the PCR products into *P. aeruginosa* PAO1 cells harboring the helper plasmid pBBR1-Rha-redγ-BAS-kan was performed using an Eppendorf electroporator. Positive clones were selected on LB agar containing 25 μg/ml gentamicin (Sangon, Shanghai, China) and confirmed via PCR. Cre recombinase-mediated excision of the antibiotic resistance gene was induced by isopropyl-D-1-thiogalacto-pyranoside (IPTG), and the loss of the resistance gene in the secondary recombinants was further confirmed by PCR.

The pHERD20T-*oprD* construct was created using Gibson assembly [31], connecting the wild-type *oprD* gene to the pHERD20T vector. The pHERD20T-*oprD* and the empty pHERD20T plasmid were electroporated into both PAO1 and 11 CROPA strains. Positive clones were identified by culturing in media containing carbenicillin and confirmed through PCR amplification.

The pHERD20T-*oprD* and pHERD20T strains were cultured in MH medium containing 200 μg/mL carbenicillin and 0.2% L-arabinose. Minimum Inhibitory Concentration (MIC) of imipenem and meropenem were determined to evaluate the antibiotic sensitivity of strains overexpressing OprD. Strain harboring the empty plasmid pHERD20T was established as a control.

### Outer membrane protein extraction

Wild-type PAO1 and CROPA strains were cultured in LB medium without antibiotics. Transformed pHERD20T-*oprD* and pHERD20T strains were grown in LB medium supplemented with 100 μg/ml carbenicillin and 0.2% L-arabinose. All cultures were incubated at 37 °C with shaking (200 rpm) until reaching an optical density (OD_600_) of 1.0.

Cells were harvested by centrifugation at 3,700 rpm for 15 min at 4 °C. The pellet was resuspended in 10 mM Tris-HCl lysis buffer (pH 7.0) and homogenized using an ultrasonic disruptor at 4 °C. The lysate was centrifuged at 1,600 ×g for 10 min at 4 °C, followed by centrifugation of the supernatant at 21,500 ×g for 30 min at 4 °C. The resulting pellet was solubilized in 10 mM PBS (pH 7.4) containing 20 g/L dodecyl maltoside, incubated at room temperature for 30 min, and centrifuged at 20,500 ×g for 40 min at 4 °C. Finally, the purified outer membrane proteins were resuspended in 10 mM PBS (pH 7.0) and total protein concentration was determined in triplicate using BCA assay. Samples were then adjusted to equal concentrations with lysis buffer prior to mixing with loading dye and analyzed by SDS-PAGE. OprD preparation followed established protocols [32].

### Analysis of mutant amino acids based on OprD structure

The OprD structural model (PDB 3SY7) was visualized in PyMOL v2.2.2 after removing solvent molecules. Mutation sites were mapped using clinical isolate sequencing data and rendered as orange spheres against a blue cartoon backbone.

### Literature review methodology

A systematic literature search was conducted in PubMed using the keywords “carbapenem-resistant *Pseudomonas aeruginosa*”, “MDR-CRPA” combined with Boolean operators (OR). Studies published between 2000 and 2024 were screened for relevance based on predefined inclusion criteria (clinical isolates, resistance mechanism) and exclusion criteria (reviews, non-human studies). Data extraction focused on specific variables (resistance rates, genetic mutations, carbapenemase, efflux pump) which were harmonized using standardization method, e.g., CLSI MIC breakpoints. Comparative analyses were performed to contextualize findings against existing evidence.

## Result

### Antimicrobial susceptibility profiles and STs of CROPA

**Fig. 1:**
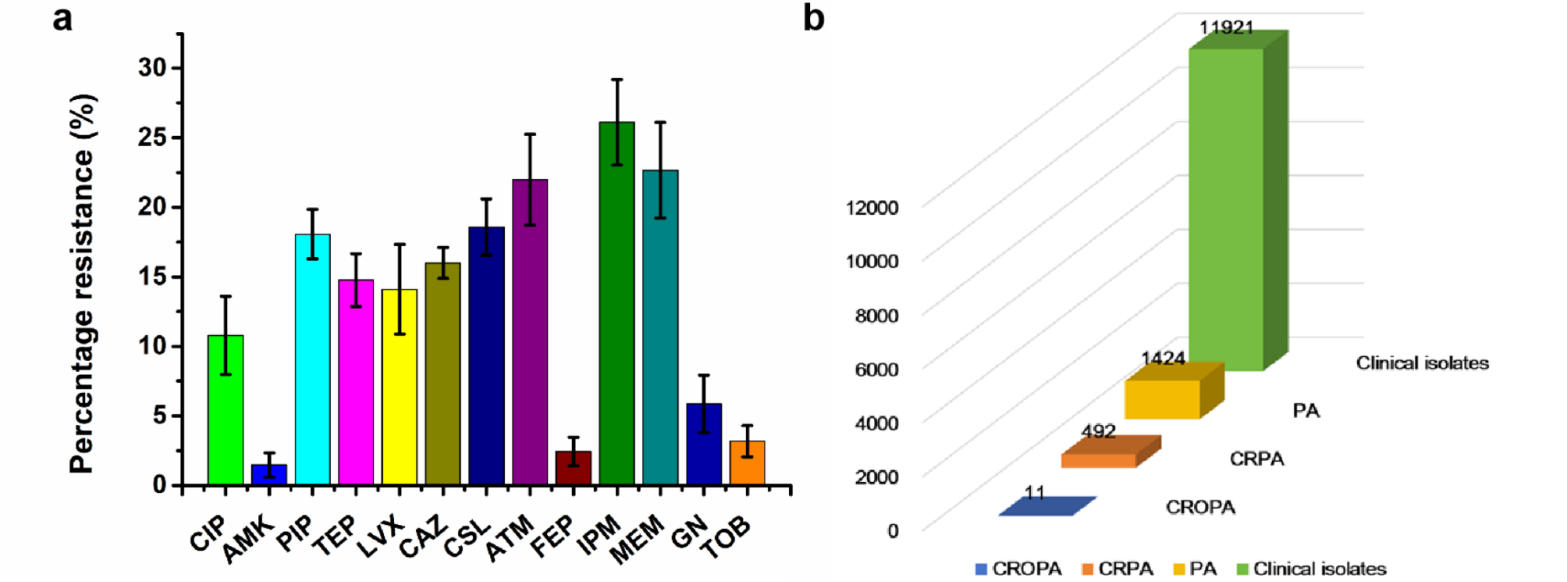
Antibiotic resistance analysis of clinical isolates. (**a**) The resistance rates of clinically isolated *P. aeruginosa* to commonly used antibiotics in clinical practice. Antibiotic resistance rates were calculated annually from 2021 to 2023. The final values represent three-year averages with error bars indicating standard deviation across biological years. We have observed that the resistance rate of *P. aeruginosa* to the carbapenem antibiotic imipenem reaches as high as 26.11%, and the resistance rate to meropenem has reached 22.67%. Note: ciprofloxacin (CIP), amikacin (AMK), piperacillin (PIP), teicoplanin (TEP), levofloxacin (LVX), ceftazidime (CAZ), cefoperazone/sulbactam (CSL), aztreonam (ATM), cefepime (FEP), imipenem (IPM), meropenem (MEM), gentamicin (GN), tobramycin (TOB). (**b**) The number of clinically isolated strains. The figure illustrates 11,921 clinical isolates, comprising 1,424 *P. aeruginosa*, 492 CRPA, and 11 CROPA strains

During the period spanning 2021 to 2023, a total of 11,921 clinical bacterial isolates were obtained from a tertiary hospital, among which 1,424 were identified as *P. aeruginosa*. Subsequent antibiotic susceptibility testing of these *P. aeruginosa* isolates indicated a notable resistance profile to commonly prescribed antibiotics. Notably, a significant proportion displayed heightened resistance towards carbapenems such as imipenem and meropenem, with resistance levels approaching 30% (Fig. 1a). Descriptive analysis revealed that among 492 carbapenem-resistant *P. aeruginosa* strains, only 11 (2.2%) isolates were classified as CROPA (Fig. 1b).

Table 1 presents an analysis of the patient profiles of the 11 isolated CROPA strains, including 7 males and 4 females, predominantly isolated from patients’ sputum and bronchoscope aspiration samples. Except for patients with COP, another ten elderly patients were aged 63 and above with underlying chronic conditions. Additionally, 6 patients had fatal outcomes, while the outcomes of the remaining patients who were discharged were not tracked. Through the analysis of microbiological test results throughout the entire hospitalization process of patients, Patient CROPA-10 was detected with the only strain of *P. aeruginosa* and it was CROPA and treatment with piperacillin / tazobactam was successful. Among the other 10 patients who were hospitalized multiple times due to various diseases, different drug-resistant characteristics of *P. aeruginosa* were detected. Even on the same day, different drug-resistant strains of *P. aeruginosa* were found in different test sites, for instance, CROPA in sputum and MDR-CRPA in urine. This also implies that eradicating *P. aeruginosa* infections completely is difficult and requires medication based on the drug resistance analysis results of susceptible areas in the body.

**Table 1:** Clinical characteristics of 11 patients infected/colonized with CROPA strains

Patient	Gender	Age (years)	Clinical specimen	Comorbidities	Outcome
01	M	75	sputum	PNA, COPD	Death
02	M	69	bronchoscope aspiration	PNA, CI	Death
03	F	78	sputum	PNA, Bronchiectasia	ND
04	F	90	sputum	PNA, HTN	Death
05	M	92	sputum	PNA, SSTI	Death
06	F	68	bronchoscope aspiration	PNA, CARD	ND
07	M	88	sputum	PNA, IHD	ND
08	M	87	sputum	PNA, IAI	ND
09	M	63	sputum	CI, T2DM	Death
10	F	53	sputum	COP, PNA	ND
11	M	93	sputum	PNA, CARD	Death

Note: The following abbreviations correspond to comorbidities/conditions analyzed in this study: Chronic Obstructive Pulmonary Disease (COPD), Pneumonia (PNA), Cerebral Infarction (CI), Type II Diabetes Mellitus (T2DM), Bronchiectasis, Hypertension (HTN), Skin and Soft Tissue Infections (SSTI), Ischemic Heart Disease (IHD), Intra-abdominal Infection (IAI), Carbon Monoxide Poisoning (COP), Cardiovascular Disease (CARD). ND (not detected) indicates that the outcome was not detected in the study

**Fig. 2:**
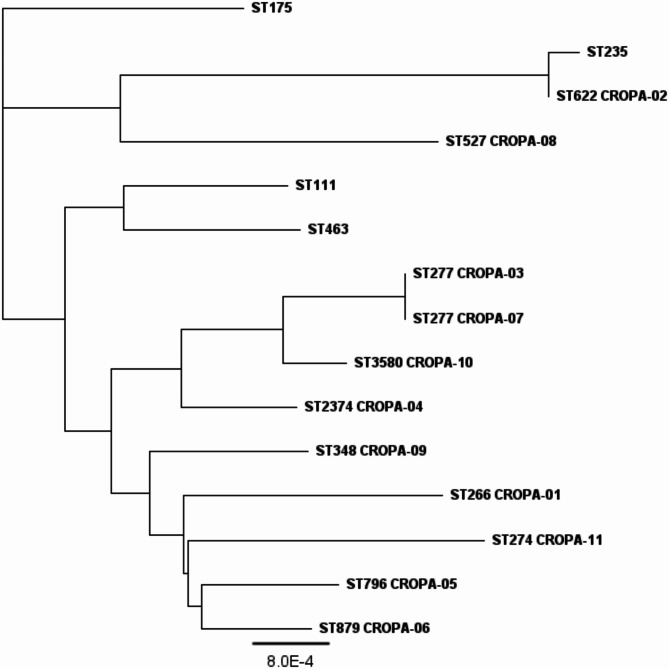
Molecular phylogeny of 15 strains of *P. aeruginosa*. A maximum likelihood phylogenetic tree was constructed from the genomes of 11 CROPA strains

After completing the genomic scan of the CROPA strain, and conducting MLST analysis. The core objectives of MLST analysis in this study were genetic specificity validation: To confirm that the 11 CROPA clinical isolates represent distinct genetic clones rather than repeated sampling of a single epidemic strain. Additionally, comparative analysis was performed with several globally prevalent highly virulent resistant strains, including ST111, ST175, ST235, ST463, to establish their phylogenetic relationships. The resulting evolutionary tree, as depicted in Fig. 2, illustrates the close relationships within the population of the 11 CROPA strains. Notably, CROPA-03 and CROPA-07 were grouped under ST277 on a single branch, while CROPA-02 showed a closer phylogenetic proximity to ST235, a common highly virulent strain.

Based on the whole-genome sequencing results, further analysis of several toxin was conducted, with the findings detailed in Table 2. It was observed that only CROPA-02 harbors the *exoU* gene, which is associated with high virulence [33].

**Table 2:** Analysis of toxin of 11 CROPA strains

Strains	*exoU*	*exoS*	*exoT*	*exoY*
CROPA-01	-	+	+	+
CROPA-02	+	-	+	+
CROPA-03	-	+	+	+
CROPA-04	-	+	+	+
CROPA-05	-	+	+	+
CROPA-06	-	+	+	+
CROPA-07	-	+	+	+
CROPA-08	-	+	+	+
CROPA-09	-	+	+	+
CROPA-10	-	+	+	+
CROPA-11	-	+	+	+

Note: “+” denotes existence, and “-” indicates non-existence

### Carbapenemases and overexpression of efflux pumps are absent in CROPA

The *bla*_VIM_, *bla*_IMP_, *bla*_KPC−2_, *bla*_NDM−1_, and *bla*_OXA−48_ genes were not identified, and there were no issues detected with the DNA template when using the *rpsL* gene for testing. This indicates that the CROPA strains do not possess common carbapenemases.

To determine whether the resistance to carbapenems is associated with reduced antibiotic accumulation due to efflux, RT-qPCR was employed to assess the expression of *mexA*, *mexC*, *mexE*, and *mexX* in each group of cells. Although there were some mutations in the transcription factors of efflux pumps, analysis revealed that these mutations had been reported by researchers previously [34, 35]. No overproducing of *mexA*, *mexC*, *mexE*, or *mexX* was observed in any CROPA strains (Table S3).

These findings suggest that in *P. aeruginosa*, the development of carbapenem resistance may not necessarily rely on the overproducing of efflux pumps.

### Decreased expression or termination prematurely of OprD in CROPA

**Fig. 3:**
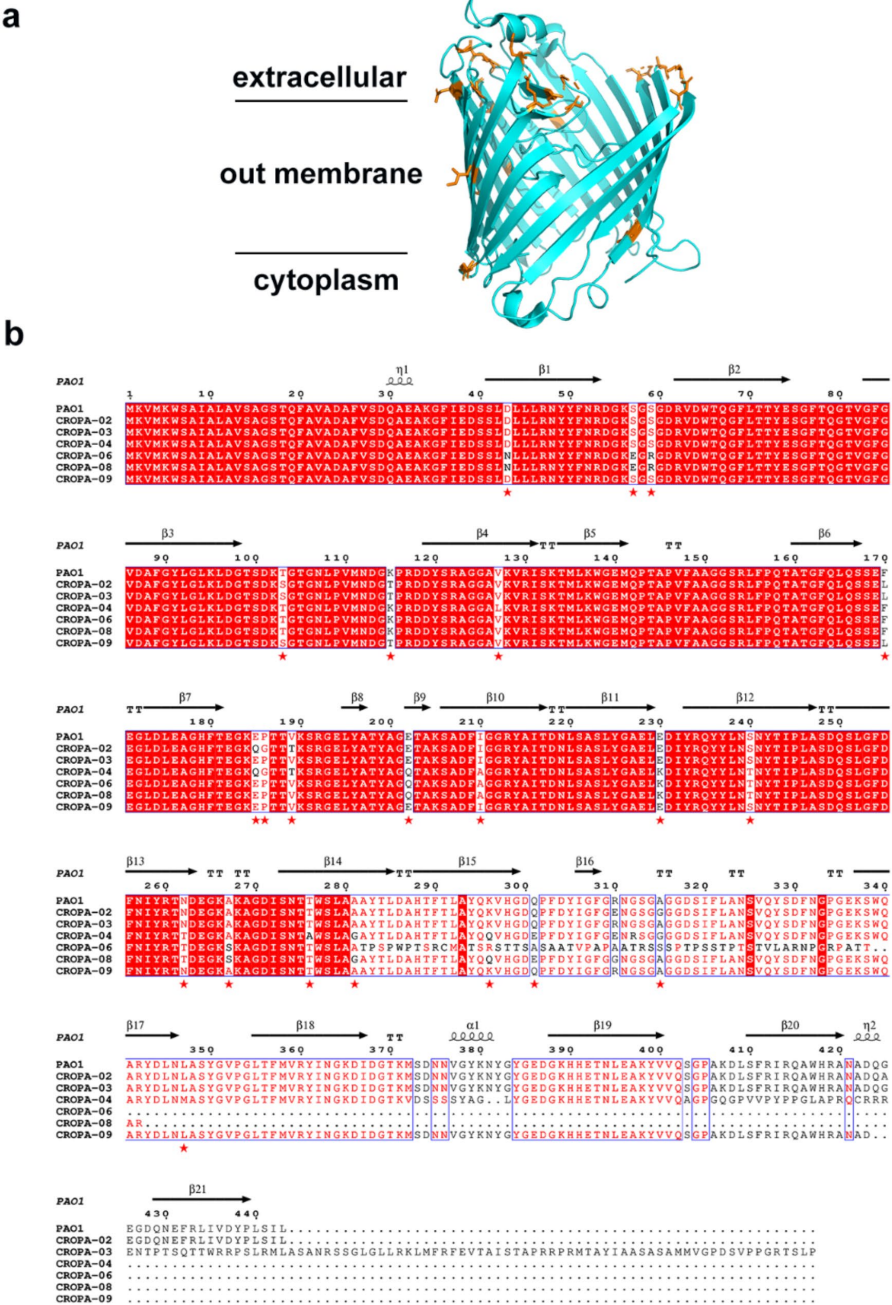
Analysis of OprD mutations in amino acids. (**a**) Analysis of mutant amino acids based on OprD structure. The figure indicates the extracellular, intracellular, and outer membrane regions. The amino acids highlighted in orange represent the mutated amino acids in the summarized CROPA’s OprD. (**b**) Alignment of amino acid variation based on OprD sequence. Scale numbers above of the alignment refer to amino acid positions in variant OprD β-Strands (according to PDB structure 3SY7) are underlined. CLUSTALW (https://www.genome.jp/tools-bin/clustalw) and ESPript 3.0 (http://espript.ibcp.fr/ESPript/cgi-bin/ESPript.cgi) were used to compare OprD sequence of PAO1 with CROPA-02, CROPA-03, CROPA-04, CROPA-06, CROPA-08, CROPA-09. Residues different from PAO1 OprD are marked with red pentagons

Using the antibiotic pan-sensitive strain PAO1 which is sensitive to all clinically detected antibiotics as the reference genome, we conducted a mutation site analysis of the *oprD* gene and identified mutations in the *oprD* gene in all 11 CROPA strains. The amino acid sequence alignment of OprD between CROPA and PAO1 has been conducted, and the alignment results are presented Fig. 3b. The main sequence alignment focused on the amino acid mutation status of CROPA-02, CROPA-03, CROPA-04, CROPA-06, CROPA-08, and CROPA-09. In addition, 8 CROPA strains exhibited premature termination of *oprD* gene expression, as outlined in Table 3. Due to the deletion of amino acids 1–82 in CROPA-07, and terminated prematurely of the 228th amino acid translation of the OprD of CROPA-05 the terminated prematurely of the 110th amino acid translation of the OprD of CROPA-10, terminated prematurely of the 73th amino acid translation of the OprD of CROPA-11, no above amino acid sequence alignment with PAO1 was conducted. In CROPA-01, an insertion of a 1,350-base pair sequence identified as belonging to the IS256 element occurs after the 187th base of the *oprD* gene. IS256 element is often associated with antibiotic resistance genes, facilitating the spread of resistance genes within bacterial populations, leading to the development and dissemination of resistance [36]. The DNA sequence of CROPA-01 is provided in the supplementary materials.

Through structural analysis of OprD (Fig. 3a), it was observed that multiple amino acid mutations predominantly occurred in regions external to the antibiotic contact sites and within irregularly folded positions. When compared with reported MDR CRPA strains, similar amino acid mutations were detected in OprD [37, 38].

**Table 3:** Mutations in OprD and down-regulated levels of *OprD* mRNA

Strains	Amino acid changes	Position of premature termination	Insertion sequence	mRNA relative down-regulated level
CROPA-01	D43N, S57E, S59R, W55-G90	none	IS256	65.66
CROPA-02	T103S, K115T, F170L, E185Q, P186G, V189T, R310E, A315G, G425A	none	none	118.89
CROPA-03	T103S, K115T, F170L	427STOP	none	291.52
CROPA-04	V127L, E185Q, P186G, V189T, E202Q, I210A, E230K, S240T, N262T, T276A, A281G, K296Q, Q301E, R310E, G312R, A315G, L347M, S373Q deletions, 375–383, S403A, P405-G425	426STOP	none	0.21
CROPA-05	D43N, S57E, S59R, E202Q, I210A	228STOP	none	383.01
CROPA-06	D43N, S57E, S59R, E202Q, I210A, E230K, S240T, N262T, A267S, A281-K338	339STOP	none	0.41
CROPA-07	1–82 deletions	none	none	4.56
CROPA-08	D43N, S57E, S59R, E202Q, I210A, E230K, S240T, N262T, A267S, A281G, K296Q, Q301E, R310G	343STOP	none	1.43
CROPA-09	T103S, K115T, F170L	424STOP	none	0.38
CROPA-10	T103S, P109Q	110STOP	none	3.3
CROPA-11	D43N, S57E, S59R	73STOP	none	59.93

Furthermore, qPCR was utilized to assess the transcription levels of the *oprD* gene in the 11 CROPA strains. The study revealed that in 7 of the CROPA strains, the transcriptional level of OprD was reduced by several dozen- to hundred-fold (Table 3).

**Fig. 4:**
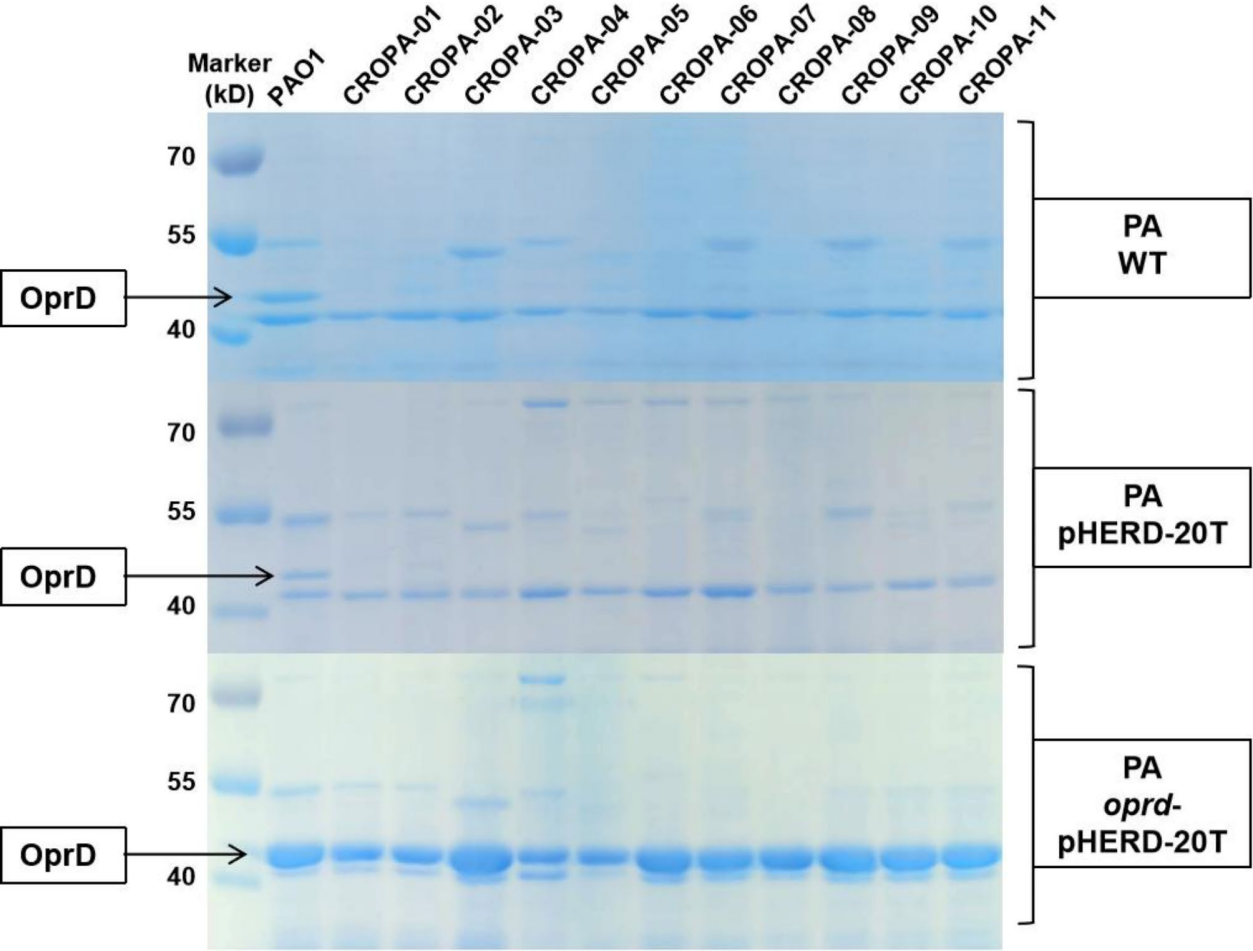
The SDS-PAGE results of OprD protein expression. The arrows indicate the position of the OprD band. In the wild-type *P. aeruginosa* (WT PA) and the empty vector control (pHERD-20T) PA, OprD expression was only observed in PAO1. Following the introduction of *oprD*-pHERD-20T, all PA exhibited overexpression of OprD

Extracting the outer membrane proteins of CROPA with PAO1 as a control revealed a weakening or absence of the band corresponding to OprD (molecular weight 46 kD) between 40 and 55 kD, indicating a reduction in OprD expression in CROPA. The band indicated by the arrow in the PAO1 lane of Fig. 4 was subjected to mass spectrometry (MS) analysis to confirm the identity of OprD. MS results verified that the band corresponds to OprD. The MS data for the three experimental conditions—PAO1, PAO1 pHERD-20T, and PAO1 *oprd*-pHERD-20T (arrows in Fig. 4) — are provided in the Supplementary Materials.

### The overexpression of wild-type OprD made CROPA sensitive to imipenem

To confirm that loss of OprD function confers resistance to carbapenem antibiotics, an *oprD* knockout strain was constructed. The MIC of imipenem increased from 2 mg/L to 32 mg/L following OprD deletion, while the MIC of meropenem rose from 1 mg/L to 4 mg/L, demonstrating enhanced antibiotic resistance. After successfully introducing the wild-type *oprD* gene into the CROPA strain, an antibiotic resistance analysis was conducted. Plasmid expression was induced by adding carbenicillin and L-arabinose in MH medium, which affects carbapenem resistance detection. Therefore, only a comparison of MIC between *oprD*-pHERD20T and pHERD20T empty vectors was performed, and resistance or sensitivity cannot be determined solely based on the MIC values obtained. As shown in Table 4, the CROPA strains with the *oprD* gene inserted exhibited a decrease in MIC for imipenem and meropenem ranging from 2 to 64 fold, indicating increased antibiotic sensitivity. The sensitivity levels were consistent with those observed when the *oprD* gene was introduced into the PAO1 strain, and in some cases, even more sensitive. Furthermore, extraction of outer membrane proteins via SDS-PAGE confirmed successful overexpression of OprD as shown in Fig. 4. This suggests that it is indeed the presence of wild-type OprD that makes *P. aeruginosa* more sensitive to carbapenem.

Due to the presence of not only OprD but also other entry points for meropenem, the resistance of CROPA to meropenem is not significantly strong when OprD is inactive. However, the overexpression of wild-type OprD has led to some CROPA strains exhibiting up to a 32 - fold increase in sensitivity to meropenem, indicating that the overexpression of OprD complements the transport pathway for meropenem, thereby enhancing antibiotic sensitivity.

Our analysis primarily focused on the impact of overexpressing wild-type OprD on the resistance to imipenem, which relies solely on OprD as its entry point. From the MIC results, we observed that CROPA showed an enhanced sensitivity to imipenem ranging from 4 to 64 times. This raises the question: what causes the discrepancies in the fold changes? SDS-PAGE results indicated that while CROPA-05 exhibited the lowest level of overexpression among the CROPA strains, its sensitivity was enhanced by 32 times, suggesting that the level of OprD overexpression does not significantly influence resistance changes.

Considering that the differences in sensitivity post OprD overexpression were not substantial, with 8 out of 11 MIC levels reaching the OprD overexpression level of PAO1, the primary determinant remains the baseline resistance level of CROPA. The strain showing a 64 - fold increase in sensitivity had an OprD1-82 amino acid deletion (CROPA-07) and premature termination of OprD73 expression (CROPA-11). Those exhibiting a 32 - fold increase in sensitivity included CROPA-05 with premature termination of OprD228 and CROPA-06 with premature termination of OprD339. The four CROPA strains with the most significant MIC changes all had large OprD fragment deletions. Despite CROPA-06, CROPA-09, CROPA-04, and CROPA-08 not showing reduced OprD transcription levels, their MIC values changed by 32, 16, and 8 times, respectively. Analysis of these representative strains suggests that changes in OprD transcription levels did not play the most critical role. Excluding the impact of transcriptional downregulation, CROPA-02 exhibited a 16-fold change in MIC due to amino acid mutations.

Our results suggest that the deletion of large OprD fragments or amino acid mutations are more likely to induce resistance to imipenem compared to downregulation of transcription levels. The knockout of OprD in PAO1 directly confirms its role in imipenem resistance.

**Table 4:** The MIC (mg/L) of *P. aeruginosa* for imipenem and meropenem

MIC (mg/L)	pHERD20T IPM	*oprD*-pHERD20T IPM	Fold Change	pHERD20T MEM	*oprD*-pHERD20T MEM	Fold Change
PAO1	1	0.5	2	2	0.5	4
CROPA-01	8	0.5	16	2	0.5	4
CROPA-02	8	2	4	2	1	2
CROPA-03	8	0.5	16	4	0.5	8
CROPA-04	8	1	8	4	2	2
CROPA-05	16	0.5	32	8	0.5	16
CROPA-06	16	0.5	32	4	0.5	16
CROPA-07	16	0.25	64	2	0.5	4
CROPA-08	8	1	8	2	0.5	4
CROPA-09	8	0.5	16	2	< 0.125	> 16
CROPA-10	8	0.5	16	0.5	< 0.125	> 4
CROPA-11	16	0.25	64	4	< 0.125	> 32

Note: Abbreviations in the table are listed here. The pHERD20T IPM represents the MIC against IPM after overexpression of the pHERD20T plasmid in *P. aeruginosa*. The *oprD*-pHERD20T IPM represents the MIC against IPM after overexpression of the *oprD*-pHERD20T plasmid in *P. aeruginosa*. The pHERD20T MEM represents the MIC against MEM after overexpression of the pHERD20T plasmid in *P. aeruginosa*. The *oprD*-pHERD20T MEM represents the MIC against MEM after overexpression of the *oprD*-pHERD20T plasmid in *P. aeruginosa*. Multiple of MIC represents the reduction in the MIC of *oprD*-pHERD20T over expression compared to pHERD20T for the same antibiotic

### Comparison of resistance mechanisms between CROPA and MDR-CRPA

After obtaining the above results, we conducted a comparative analysis of the resistance mechanisms of CROPA and MDR-CRPA. Starting with the three most critical mechanisms, we explored their differences and aimed to elucidate the development process from single-drug resistance to extensively drug-resistant strains. A study on MDR-CRPA revealed that 87.1% of MDR-CRPA strains exhibited OprD mutations or loss of function [39, 40], with the specific amino acid mutations detailed in Table 5. The remaining 11.9% of resistant bacteria, without OprD inactivation, displayed resistance through the production of carbapenemases or overexpression of efflux pumps at varying probabilities, making resistance more plausible [41, 42]. According to our experiments, 100% of CROPA strains had OprD mutations or loss of function. The amino acid mutations in CROPA’s OprD encompassed all mutations found in MDR-CRPA, indicating that the amino acid positions in OprD listed in the table are more prone to mutations. Unlike MDR-CRPA, all CROPA strains did not produce carbapenemases or exhibit high efflux pump expression. It is suggested that carbapenem resistance has been developed even in the absence of carbapenemase and without upregulation of Mex transcription levels. While we observed amino acid mutations in efflux pump transcription factors, we did not observe overexpression of efflux pumps. The mutations found in CROPA, such as MexR V126E and NalC G71E, S209R mutations in the transcription factors of MexAB-OprM, are similar to mutations in the transcription factors of the MDR-CRPA efflux pump. However, they do not upregulate the transcription of the efflux pump, providing information for further research into the transcriptional regulation of these efflux pumps.

**Table 5:** Frequency of resistance mechanisms and amino acid mutation sites in CROPA and MDR-CRPA

	MDR-CRPA		CROPA	
OprD mutations [39]	87.1% [40]	D43N, S57E, S59R, T103S, K115T, F170L, E185Q, P186G, V189T, E202Q, I210A, E230K, S240T, N262T, A267S, A281G, K296Q, Q301E, R310G, R310E, A315G.	100%	D43N, S57E, S59R, T103S, P109Q, K115T, F170L, V127L, E185Q, P186G, V189T, E202Q, I210A, E230K, S240T, N262T, A267S, T276A, A281G, K296Q, Q301E, R310E, G312R, A315G, L347M, G425A.
carbapenemase-producing [41]	12.7 - 81%	*bla*_GES_, *bla*_KPC_, *bla*_AIM_, *bla*_GIM_, *bla*_IMP_, *bla*_NDM_, *bla*_SPM_, *bla*_VIM_, *bla*_OXA_.	0	-
higher Mex *^1^ transcription levels [42]	28.8–72.9%	Transcription factor mutations in efflux pumps *^2^ MexR: P37R, L54P, Q55R, V126E, Q137H, G137P, C138A; NalC: G71E, S209R; NalD: T158I [43]. NfxB: C199T, C113T [35]. MexS: N249D, V104A, F253L, D44E, S60F, F185L, L270Q, C245G, A166P, S60P, L263Q; MexT: G258D, Y138D, G258D [44]. MexZ: L138R, W176S, G195E [45].	0	Transcription factor mutations in efflux pumps MexR: V126E, T130P; NalC: G71E, S209R; NalD: N130S. NfxB: -. MexS: -; MexT: P202A, I298F, D302E. MexZ: L142Q, R71W, A14V.

Note: *^1^ Mex denotes efflux pumps. *^2^ Transcription factors of efflux pumps were listed in the table, including MexR, NalC and NalD, the transcription factor of MexAB-OprM; NfxB, the transcription factor of MexCD-OprJ; MexS and MexT the transcription factor of MexEF-OprN; MexZ, the transcription factor of MexXY-OprM

## Discussion

*P. aeruginosa* is a common nosocomial pathogen in hospitals, and with the overuse of antibiotics, resistance is on the rise [46]. Despite conscious antibiotic stewardship efforts to reduce the emergence of resistant strains, resistance rates remain stubbornly high [47]. Carbapenems have been the cornerstone of anti-infective therapy since their successful development in 1979, often referred to as the “last line of defense” against infections [48]. However, CRPA still accounts for over 30% of cases. It is foreseeable that in future, the available clinical antibiotic options will be severely limited, necessitating the development of new drugs or modifications to existing antibiotics to combat resistance. Therefore, it is crucial to understand the mechanisms of resistance in addressing this challenge.

In investigating the mechanisms of resistance in CRPA strains, it is noted that these strains generally exhibit insensitivity to a broad range of drugs due to their resistance profile, often characterized by multidrug resistance. The resistance conclusions drawn from these studies are intricate [49, 50]. Summarizing the mechanisms of carbapenem resistance, these include inactivation of porins, mutations in PBPs that affect carbapenem binding, carbapenemase degradation, and efflux pumps expelling antibiotics [51]. To elucidate the specific resistance mechanisms of CRPA, focus has been directed towards the rare subset of CROPA. In a span of two years, researchers at Jinan Central Hospital isolated only 11 strains of CROPA, accounting for a mere 2.2% of all CRPA cases. Building upon the known mechanisms of MDR CRPA, this study delves into the specific resistance mechanisms of CROPA.

Upon analyzing certain mutations in PBPs proteins (encoded by the *ftsI*) of CROPA and comparing them to MDR CRPA strains, similar amino acid mutations were identified [52]. Notably, not all CROPA strains exhibited changes in PBP proteins. Consequently, the emergence of CROPA strains does not appear to be significantly related to alterations in PBP proteins. Similarly, mutation analysis of biofilm-associated genes (*wspF*, *rpoS*), lipopolysaccharide biosynthesis genes (*wapR*, *galU*), alginate synthesis gene (*algU*), and nitrogen metabolism-related gene (*rpoN*) revealed that not all CROPA strains exhibited alterations in biofilm-related or metabolic proteins. Furthermore, biofilm formation—a nonspecific process that indiscriminately impedes the penetration of diverse antibiotics—would typically contribute to multidrug resistance (MDR-CRPA) rather than selective carbapenem resistance. Therefore, the emergence of CROPA strains appears unrelated to modifications in biofilm synthesis or metabolic proteins.

The most common carbapenemase genes in CRPA were tested, but none were detected. This absence is deemed reasonable, as the presence of carbapenemases would likely confer resistance to non-carbapenem antibiotics. Mentioned in the literature many carbapenemases confer resistance not only to carbapenems, but also to other β-lactam drugs, including some novel β-lactam–β-lactamase inhibitors [53]. Carbapenemase-producing CRPA isolates were less likely to be susceptible to cefepime, ceftazidime, piperacillin-tazobactam, ciprofloxacin and amikacin than non-carbapenemase-producing CRPA isolates [54].

Efflux pumps expelling antibiotics are crucial contributors to resistance [55]. Relevant efflux pumps associated with carbapenem resistance include MexAB-OprM, MexCD-OprJ, MexEF-OprN, and MexXY-OprM, regulated by multiple transcription factors such as MexR, NalC, NalD, NfxB, NfxC, MexS, and MexZ [35, 43, 56, 57]. Mutations in these transcription factors were found to be similar to those reported in other studies [58], and none of these efflux pumps exhibited transcriptional upregulation. This suggests that the detected mutations in these transcription factors did not impact efflux pump function significantly. Consequently, it is inferred that the presence of CROPA strains is not strongly associated with efflux pumps.

Finally, focusing on the changes in the outer membrane porin protein OprD. This study demonstrates that the functional loss of the OprD porin is the central mechanism driving carbapenem resistance in CROPA strains. The inactivation of OprD arises from four distinct molecular mechanisms: (1) Large Deletions or Premature Translation Termination: Premature termination of OprD translation was observed in 8 strains (confirmed by SDS-PAGE showing absence of wild-type protein). In addition, the deletion of residues 1–82 in CROPA-07 disrupted the β-barrel structure essential for channel formation. (2) Insertion Sequence Disruption: The novel integration of the IS256 element into the *oprD* gene (a previously unreported mechanism in P. aeruginosa) caused structural abnormalities in extracellular loop regions, likely impairing drug binding. (3) Critical Site Mutations: Previously reported missense mutations clustered in extracellular irregular loops may hinder antibiotic permeation through steric hindrance or electrostatic repulsion. (4) Transcriptional Dysregulation: CROPA-02 exhibited a drastic 118.89-fold reduction in *oprD* transcription and loss of protein expression. Despite an intact coding sequence, promoter defects or post-transcriptional regulation likely abolished porin production, blocking antibiotic influx. Complementation with wild-type OprD fully restored imipenem susceptibility in all strains, excluding contributions from other resistance mechanisms (e.g., efflux pumps or β-lactamases). Notably, the distinct resistance mechanisms in CROPA-02 (transcriptional silencing) and CROPA-07 (structural disruption) highlight the diversity of OprD inactivation pathways.

In conclusion, OprD dysfunction—whether through structural destruction or expression loss—serves as both a necessary and sufficient condition for CROPA resistance. The absence of MDR phenotypes in these strains further supports the exclusivity of OprD-mediated mechanisms, as concurrent resistance factors would typically generate broader MDR profiles.

Our findings should be interpreted in the context of the following limitations. First, the cohort size (*n* = 11 CROPA strains) reflects the inherent rarity of this resistance phenotype in our clinical setting. While all isolates were prospectively collected over three years from a tertiary hospital serving a large urban population (> 20 million), the low incidence (3–4 cases annually) inherently restricts sample accrual. Although the consistent OprD inactivation patterns across all strains (structural mutations or transcriptional silencing) strongly suggest biological validity, larger multi-center studies are required to confirm the generalizability of these mechanisms to other geographic or institutional settings. Second, while comprehensive screening excluded known carbapenemases (e.g., KPC, NDM, VIM) and efflux pump upregulation (e.g., *mexAB-oprM*), we cannot rule out contributions from uncharacterized resistance determinants. For example, rare β-lactamases or novel efflux systems not targeted in our assays might theoretically coexist with OprD loss. Nevertheless, the complete restoration of imipenem susceptibility upon OprD complementation in all strains strongly supports the primacy of porin dysfunction as the dominant resistance driver in this cohort.

The emergence of CROPA has significant implications for treatment. Since CROPA exhibits resistance only to carbapenems, unlike MDR CRPA, it still retains sensitivity to other antibiotics. This provides an opportunity for treatment by selecting antibiotics that are effective against CROPA without the need for broader-spectrum antibiotics. However, it is important to note that the emergence of CROPA may pave the way for the development of further resistance. If these strains are exposed to other antibiotics, especially broad-spectrum antibiotics or prolonged use of carbapenems, they may gradually develop resistance to other antibiotics, eventually evolving into MDR CRPA. Therefore, early identification and effective management of CROPA strains are crucial to prevent their transition into more challenging multidrug-resistant strains, ensuring the efficacy of treatment and patient safety. This study confirmed that OprD is the main cause of the clinical emergence of CROPA, and future antimicrobial means targeting OprD should be developed.

## Conclusion

Our study elucidated the genetic basis of carbapenem resistance in CROPA strains and compared it with previous studies on MDR-CRPA, highlighting *oprD* gene mutations as a key factor in CROPA production. Restoring wild-type *oprD* gene expression successfully reversed resistance, emphasizing its crucial role in carbapenem susceptibility. This underscores the importance of targeted interventions against CRPA infections, focusing on OprD as a potential therapeutic target to enhance treatment efficacy.

## Electronic supplementary material

Below is the link to the electronic supplementary material.

Supplementary Material 1

Supplementary Material 2

Supplementary Material 3

## Data Availability

The datasets generated or analysed during the current study are available in the NCBI’s Sequence Read Archive (SRA), under BioProject accession number PRJNA1191633.
