# Supplementary material for: Loss of OprD function is sufficient for carbapenem-resistance-only but insufficient for multidrug resistance in Pseudomonas aeruginosa
Source: BMC Microbiol. 2025 Apr 16;25:218. doi: 10.1186/s12866-025-03935-3 (PMC12001449; doi:10.1186/s12866-025-03935-3)
Supplement: Supplementary file 1 — Supplementary Material 1 [file 12866_2025_3935_MOESM1_ESM.docx]

>CROPA-01 oprD

ATGAAAGTGATGAAGTGGAGCGCCATTGCACTGGCGGTTTCCGCAGGTAGCACTCAGTTCGCCGTGGCCGACGCATTCGTCAGCGATCAGGCCGAAGCGAAGGGGTTCATCGAAGACAGCAGCCTGAACCTGCTGCTCCGCAACTACTATTTCAACCGTGACGGCAAGGAAGGTCGGGGTGATCGCGTCGATTGGGGCCTGTCAGTAATTTCGTGTTTGGGGCATAACATGAGGTCAAGGAGTGTCTATGTCTCGCAAACCAAGAACCCAGCCGGCAGCGTTGCCTGTGATTCCTTCCGAGTTGCTGGAGTCGTTTGGTGACGGCTCGATGACGGCCGAAGCTATCAATGCGGCGTCCCTGGCTTTCAAGAAGGCCCTGATCGAGCGGGCCCTGGCCGGTGAGCTGAACCATCACCTGGGCTACCCGGCCGGCACAGCCAAGCCCGAGCGGATGAGTAATCAGCGCAACGGCAAGGGGGCCAAGACGGTGCTGACCCAGGAGGGGCCGATCCGTATCGACGTGCCCCGAGATCGCGAGGGCAGCTTTGCCCCGCTCCTGATCCCCAAGCATGAGCGCCGTTTTACTGGCTTTGATGACAAGATCGTCGCCATGTATGCCCGCGGCATGACGGTGCGCGAAATCCAGGGCTTTCTGCTGGAACAATACGGCACAGACGTCTCGCCGGACTTCATCAGCTCGGTCACTGACGAGGTTATGGCCGAGGTCACCGCCTGGCAGGCCAGGCCGCTCGAGCCCATGTATCCGGTCGTGTTCTTTGACGCGCTGCGGGTCAAGATCAGGGAGGATGCTGTCGTGCGCAACAAGGCCGTCTATCTGGCCCTGGGCGTGCTACCCGACGGCACACGCGACATCCTGGGTTTGTGGATTGAAGGCACCGAAGGCGCCAAGCTCTGGATGAAGGTCTTCAACGACCTGAAGACCCGGGGCGTGGGCGATATCCTGATCGCCGTGACTGACGGGTTGAAGGGTATACCCGAGGCGCTGGCCGCGGTGTTCCCGGCCACCACACTGCAAACCTGCATCGTCCATCTGATCCGCAACAGCTCGATTACGCGAGCTGGAAGGATCGCAAGGCGCTGGCCGCCGCGATCCGACCCGATCTATACCGCTGTCAGTGCTGAAGCGGCCCTGGCCGCGCTTGACGCCTTTGCCGATGGGCCGTGGGGTCAGAAGTTCCCCACCGTCTGCGCGGCATGGCGCAATGCCTGGGATCGCGTGATTCCGTTCTTCGCCTTTGCGCCGGAGATCCGCAAGGTGATCTACACCACCAACGCCATCGAGAATGTCAACTCGCAGCTACGCAAGATCATCAAGACCCGGGGCCATTTCCCTACTGACGAAGCCGCCTCCAAGTTGATCTGGCTCGCGCTGCGAAACATCACCGCCAAATGGAGCCGATCCGCTCACGACTGGAAGCAAGCCATGAACCAATTCGCTATCCTTTACGCTGACCGATTCAGTCGCCCTTCCGTGTAATTCTCTACCCGCCCTAAACACGGAATTTATGACACCCCCGTCGATTGGACCCAGGGCTTCCTCACCACCTACGAATCCGGCTTCACTCAAGGCACCGTGGGCTTCGGCGTCGATGCCTTCGGCTACCTGGGCCTGAAGCTCGACGGCACCTCCGACAAGACCGGCACCGGCAACCTGCCGGTGATGAACGACGGCAAGCCGCGCGATGACTACAGCCGCGCCGGCGGCGCCGTGAAGGTGCGCATCTCCAAGACCATGCTGAAGTGGGGCGAGATGCAACCGACCGCCCCGGTCTTCGCCGCTGGCGGCAGCCGCCTGTTCCCGCAGACCGCGACCGGCTTCCAGCTGCAGAGCAGCGAATTCGAAGGGCTCGACCTCGAGGCAGGCCACTTCACCGAGGGCAAGGAGCCGACCACCGTCAAATCGCGCGGTGAGCTCTACGCCACCTACGCCGGCCAGACAGCCAAGAGCGCCGACTTCGCTGGCGGCCGCTACGCGATCACCGACAACCTCAGCGCCTCCCTCTATGGCGCAGAGCTGAAAGACATCTATCGCCAGTACTACCTGAACACCAACTACACCATCCCGCTGGCCTCCGATCAATCGCTGGGCTTCGACTTCAACATCTACCGCACCACCGACGAAGGCAAGTCCAAGGCTGGCGACATCAGCAACACCACCTGGTCCCTGGCGGGCGCGTATACCCTGGACGCCCACACCTTCACCCTGGCCTACCAGCAGGTGCATGGCGACGAGCCGTTCGACTACATCGGCTTCGGCGGCAACGGTTCCGGCGCCGGCGGCGACTCGATCTTCCTCGCCAACTCCGTCCAGTACTCCGACTTCAACGGTCCTGGCGAGAAATCCTGGCAGGCCCGCTACGACCTGAACCTGGCCTCCTACGGCGTTCCTGGCCTGACCTTCATGCTGCGTTACATCAATGGTAAGGACATCGACGGTACCAAGGTCGATTCCAGCTCCTCCTATGCAGGCCTGTACGGCGAGGATGGCAAGCACCACGAAACCAACCTCGAAGCCAAGTACGTGGTCCAGTCCGGTCCGGCCAAGGACCTGTCGTTCCGCATCCGCCAGGCCTGGCACCGTGCCAACGCCGACCAGGGCGAAGGCGACCAGAACGAGTTCCGCCTGATCGTCGACTATCCGCTGTCGATCCTGTAATCGACCGACAGGCAACGAAAAAACCCGGCATCGCCGGGTTTTTTCTTCTTGGCGGCAACGCGCCTATAAAGGAAGGGCGTAGGTACCGGTGACATGGGCCACCAGGTCTTCCTCATTACTCTGCGAAAACACTGAAA

Note: The red letter marks the inserted IS256 element sequence.
