# Supplementary material for: Loss of OprD function is sufficient for carbapenem-resistance-only but insufficient for multidrug resistance in Pseudomonas aeruginosa
Source: BMC Microbiol. 2025 Apr 16;25:218. doi: 10.1186/s12866-025-03935-3 (PMC12001449; doi:10.1186/s12866-025-03935-3)
Supplement: Supplementary file 2 — Supplementary Material 2 [file 12866_2025_3935_MOESM2_ESM.docx]

**Supplementary Data Table S1. Sequence of primer for carbapenemase gene amplification.**

| **Carbapenamases (CPM)** | **Primer sequences** | | **Product size [bp]** |
| --- | --- | --- | --- |
| VIM | F | AGTGGTGAGTATCCGACAG | 261 |
|  | R | ATGAAAGTGCGTGGAGAC |  |
| IMP | F | GGAATAGAGTGGCTTAATTCTC | 233 |
|  | R | GGTTTAACAAAACAACCACC |  |
| KPC-2 | F | TGTAAGTTACCGCGCTGAGG | 233 |
|  | R | CGGCGGCGTTATCACTGTAT |  |
| OXA-48 | F | ATGGACAGACGCGCGATATC | 321 |
|  | R | AGTCACCATTGGCTTCGGT |  |
| NDM-1 | F | GCCCAGATCCTCAACTGGAT | 286 |
|  | R | TATTGTCACTGGTGTGGCCG |  |

**Supplementary Data Table S2. RT-qPCR and *oprd* gene knockout primer sequence.**

| **Gene** | **Primer sequences** | | **Product size [bp]** |
| --- | --- | --- | --- |
| MexA RTF | F | CAAGCAGAAGGCCATCCTC | 182 |
| MexA RTR | R | CGGTAATGATCTTGTCGCCG |  |
| MexC RTF | F | AGGAAGGATCGGGGCGTTG | 148 |
| MexC RTR | R | CCCAGGCAGTTCCGAACTCA |  |
| MexE RTF | F | CTGATCAAGGACGAAGCGG | 172 |
| MexE RTR | R | GCAGGCCATTCACGACGATC |  |
| MexX RTF | F | CCTGTTCCGCAATCCGCATC | 176 |
| MexX RTR | R | GGACCTCCACGTCTTCCACC |  |
| OprD RTF | F | GAAGCCAAGTACGTGGTCCAG | 144 |
| OprD RTR | R | CAGGATCGACAGCGGATAGTC |  |
| RpsLF | F | GCAACTATCAACCAGCTGGTG | 230 |
| RpsLR | R | GCTGTGCTCTTGCAGGTTGTG |  |
| *oprD*-genta | F | cttcctttataggcgcgttgccgccaagaagaaaaaacccggcgatgccgggttttttcgttgcctgtcggtcgaAGCTGAATTACATTCCCAACCG | 750 |
| *oprD*-genta | R | gccgacaagaagaactagccgtcactgcggcactgtgatggcagagataatttcaaaaccaaaggagcaatcacaCAACTTAAATGTGAAAGTGGGTC |  |

**Supplementary Data Table S3. Mutations in efflux pump regulators and up-regulated levels of efflux pump mRNA.**

| Strains | MexR | NalC | NalD | *mexA* | MexS | MexT | *mexE* | NfxB | *mexC* |
| --- | --- | --- | --- | --- | --- | --- | --- | --- | --- |
| CRPA-01 | - | G71E、H30Y | - | 0.69 | - | P202A | 1.42 | - | 0.17 |
| CRPA-02 | V126E | G71E、S209R、E153Q | - | 0.00053 | - | P202A | 1.3 | - | 0.022 |
| CRPA-03 | - | G71E、S209R | - | 0.0057 | - | P202A、I298F | 1.22 | - | 0.21 |
| CRPA-04 | V126E | G71E、A186T | - | 1.29 | - | P202A | 0.6 | - | 0.3 |
| CRPA-05 | V126E | G71E、S209R | - | 0.69 | - | P202A | 0.59 | - | 0.36 |
| CRPA-06 | V126E | G71E、S209R | - | 0.004 | - | P202A | 1.54 | - | 0.08 |
| CRPA-07 | - | G71E、S209R | - | 0.00085 | - | P202A、I298F、D302E | 1.22 |  | 0.25 |
| CRPA-08 | - | G71E | N130S | 0.9 | - | P202A | 0.3 | - | 1.24 |
| CRPA-09 | - | G71E、S209R | - | 1.06 | - | P202A | 0.5 | - | 0.046 |
| CRPA-10 | T130P | G71E、S209R | - | 1.11 | - | P202A | 1.65 | - | 1.11 |
| CRPA-11 | - | G71E、S209R | - | 0.34 | - | P202A | 1.3 | - | 0.027 |
